# Molecular Bridge Engineering for Tuning Quantum Electronic Transport and Anisotropy in Nanoporous Graphene

**DOI:** 10.1021/jacs.3c00173

**Published:** 2023-03-29

**Authors:** César Moreno, Xabier Diaz de Cerio, Manuel Vilas-Varela, Maria Tenorio, Ane Sarasola, Mads Brandbyge, Diego Peña, Aran Garcia-Lekue, Aitor Mugarza

**Affiliations:** † Departamento de Ciencias de la Tierra y Fisica de la Materia Condensada, Universidad de Cantabria, 39005 Santander, Spain; ‡ Catalan Institute of Nanoscience and Nanotechnology (ICN2), CSIC and The Barcelona Institute of Science and Technology, Campus UAB, Bellaterra, 08193 Barcelona, Spain; ¶ Donostia International Physics Center, Paseo Manuel de Lardizabal 4, 20018 San Sebastian, Spain; § Centro de Investigación en Química Biolóxica e Materiais Moleculares (CiQUS) and Departamento de Química Orgánica, Universidade de Santiago de Compostela, 15782 Santiago de Compostela, Spain; ∥ Departamento de Física Aplicada, Universidad del País Vasco/Euskal Herriko Unibertsitatea (UPV/EHU), 20018 Donostia, Spain; ⊥ Department of Physics, Technical University of Denmark, DK-2800 Kongens Lyngby, Denmark; # Ikerbasque, Basque Foundation for Science, 48013 Bilbao, Spain; ○ ICREA − Institució Catalana de Recerca i Estudis Avançcats, Lluis Companys 23, 08010 Barcelona, Spain

## Abstract

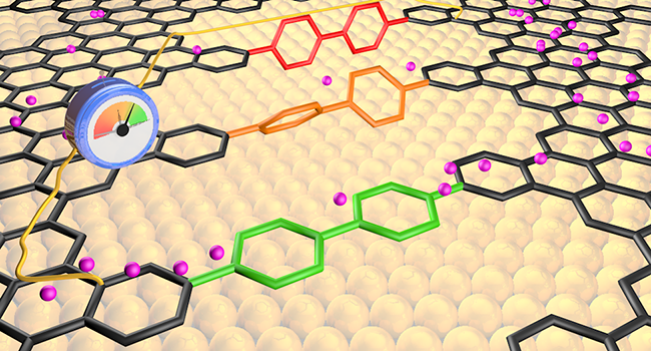

Recent advances on surface-assisted synthesis have demonstrated that arrays of nanometer wide graphene nanoribbons can be laterally coupled with atomic precision to give rise to a highly anisotropic nanoporous graphene structure. Electronically, this graphene nanoarchitecture can be conceived as a set of weakly coupled semiconducting 1D nanochannels with electron propagation characterized by substantial interchannel quantum interferences. Here, we report the synthesis of a new nanoporous graphene structure where the interribbon electronic coupling can be controlled by the different degrees of freedom provided by phenylene bridges that couple the conducting channels. This versatility arises from the multiplicity of phenylene cross-coupling configurations, which provides a robust chemical knob, and from the interphenyl twist angle that acts as a fine-tunable knob. The twist angle is significantly altered by the interaction with the substrate, as confirmed by a combined bond-resolved scanning tunneling microscopy (STM) and ab initio analysis, and should accordingly be addressable by other external stimuli. Electron propagation simulations demonstrate the capability of either switching on/off or modulating the interribbon coupling by the corresponding use of the chemical or the conformational knob. Molecular bridges therefore emerge as efficient tools to engineer quantum transport and anisotropy in carbon-based 2D nanoarchitectures.

## Introduction

At the nanoscale, even the most basic quantum size effect, the induction of semiconducting gaps by electron confinement, requires ultimate precision. The case of graphene is a dramatic example where deviations of a single atom in width can induce dramatic variations of the gap of up to a factor of 4.^1,2^ As a consequence, local defects or variations in width can severely disrupt electron transport properties in nanoscale graphene nanoribbons (GNRs).^3,4^ Fortunately, the atomic engineering of quantum phenomena in graphene-based nanomaterials started to be a reality a decade ago with the emergence of the bottom-up on-surface synthesis (OSS).^5−14^ In addition to producing atomically precise homogeneous 1D nanostructures, OSS-based methods can also introduce heteroatoms,^15,16^ heterojunctions,^17,18^ or hybrid components^19,20^ in the structure with the same precision, allowing for a precise engineering of the electronic properties.

Recently, an OSS synthetic approach based on the lateral coupling of parallel aligned nanoribbons has endowed this atomic engineering capability to the synthesis of two-dimensional nanoarchitectures that can be conceived as nanoporous graphene (NPG).^21−23^ In these intrinsically anisotropic structures, when adjacent ribbons are not equivalent, band mistmatch can lead to a total confinement of electrons in individual GNRs.^23^ In contrast, the lateral coupling of equivalent GNRs can lead to sizable interribbon transmission, giving rise to intriguing quantum interferences that regulate the degree of anisotropy of the nanomaterial.^24^ Quantum simulations on electron propagation in different proposed NPG nanoarchitectures indicate that the interribbon transmission can be switched on/off by the chemical modification of the coupling bridges.^25,26^

In this work, we present the synthesis of a new nanoporous graphene (NPG) structure where such chemical knobs are introduced and where the interribbon coupling strength can be additionally modulated by a continuous conformational transformation of the molecular bridges. The multiple bonding configurations of the bisphenylene bridges that bind the nanoribbons in this NPG can efficiently switch the interribbon electron flow off by incorporating *meta* bonds. In the *para* configuration, the interribbon electron flow is modulated by controlling the π–π overlap via the interphenyl twist angle. The capability of manipulating the interphenyl twist angle is experimentally inferred from the subtle interplay between the steric hindrance and substrate interactions that leads to different twist angles in individual ribbons and NPGs with different bridge configurations. This is further corroborated by ab initio calculations where the effect of substrate interaction is directly addressed and the energetics of the twist angle are quantitatively analyzed.

## Results and Discussion

**Figure 1:**
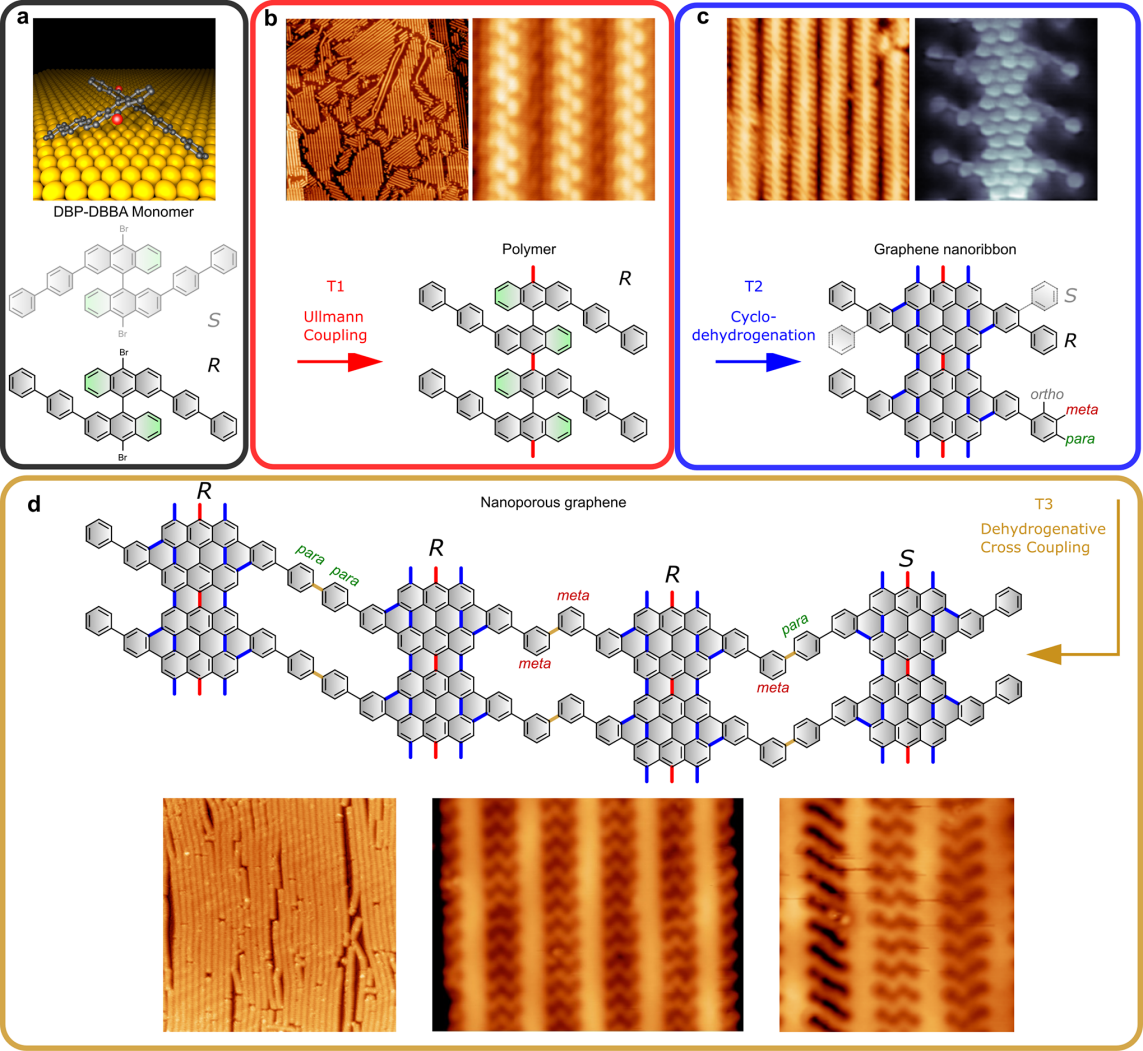
Schematic illustration and STM images of the synthetic steps for the generation of phenylene-bridged NPG. (a) Molecular structure of the DBP-DBBA precursor. (b) Self-assembled arrays of linear polymer chains obtained after the Ullmann coupling reaction induced at step *T*_1_ = 200 °C. Image size: 150 × 150 nm^2^ (left); 6.3 × 6.3 nm^2^ (right). (c) Planarization of the polymers into phenylated GNRs obtained by triggering cyclodehydrogenation at step *T*_2_ = 400 °C. The phenylene side groups and 7-13-AGNR backbone structure are resolved in the BR-STM image shown in grayscale. Image size: 20 × 20 nm^2^ (left); 3 × 3 nm^2^ (right). The two possible prochiral configurations are indicated in one of the unit cells as *S* and *R*. (d) Formation of NPG by the lateral fusion of GNRs achieved by inducing interribbon dehydrogenative cross coupling at the final step *T*_3_ = 450 °C. The chirality *S*/*R* of each GNR is indicated on top. The STM images at the bottom show large scale NPG domains (left), a pure *meta–meta* domain (center), and a mixture of the three bridge configurations (right). Image size: 98.3 × 98.3 nm^2^ (left), 13.4 × 10 nm^2^ (center), and 8.5 × 8.5 nm^2^ (right).

The building block of the on-surface synthesized nanoporous graphene is 2,2′-di([1,1′-biphenyl]-4-yl)-10,10′-dibromo-9,9′-bianthracene (DBP-DBBA), a bisanthracene derivative precursor that has been synthesized in solution following a procedure similar to that reported for another phenylated derivative^21^ (see Section 1.1 of the Supporting Information for more details on the synthesis). The steric repulsion between the anthracene units confers to the molecule the 3D staggered chiral conformation depicted in Figure 1a, giving rise to the two possible enantiomers labeled as *R*/*S*. The OSS reaction procedure carried out in ultrahigh vacuum conditions, summarized in Figure 1, starts with the deposition of the DBP-DBBA precursor on the Au(111) surface held at room temperature. By a subsequent annealing to *T*_1_ = 200 °C, biradicals generated by the thermal debromination cross-couple to form 1D polymeric chains following the well-known Ullmann coupling reaction. The DBP-DBBA units within the polymer chain retain the staggering of their bisanthracene core, conferring a double protrusion zigzag backbone appearance in the scanning tunneling microscope (STM) images, as shown in the close-up image of Figure 1b. Polymer chains self-align in homochiral close-packed islands following the three equivalent orientations imposed by the hexagonal lattice of the underlying Au(111) surface, as shown in the larger scale image of Figure 1b left (see Figure S6 for more details). A second annealing step at *T*_2_ = 400 °C converts the polymers into graphene nanoribbons by undergoing an internal cyclodehydrogenation that planarizes the structure into the hexagonal lattice of aromatic rings characteristic of graphene. The inner phenylene rings of the peripheral bisphenyl groups fuse into the aromatic backbone giving rise to a periodic modulation of the ribbon width of alternating pairs of 7 and 13 C atoms, a structure labeled as 7-13-AGNR in previous studies.^21,27,28^ The outermost phenyl rings, on the other hand, remain as single bonded peripheral phenyl groups, as can be appreciated in the bond-resolved (BR) STM image of Figure 1c, resulting in a phenylated GNR that we label as *Ph*-7-13-AGNR. As opposed to the 7-13-AGNR, where the chiral information on the precursor is lost upon cyclodehydrogenation, *Ph*-7-13-AGNR retains a prochiral configuration (i.e., chiral when confined in 2D) thanks to the side phenyl substituents. We note that the phenyl side group does not affect the band gap, which amounts to ca. 1.0 eV for of the *Ph*-7-13-AGNR (see Figure S7), essentially the same value as for the 7-13-AGNR.^21^ This is in good agreement with the band structure calculated by density functional theory (DFT), where the band gap of 0.73 eV obtained for *Ph*-7-13-AGNR varies little from the 0.74 eV obtained for 7-13-AGNR. The GNRs remain aligned in homochiral clusters that follow the three equivalent orientations of the atomic lattice of the underlying substrate, which enables their lateral coupling by triggering a dehydrogenative cross coupling after increasing the temperature to *T*_3_ = 450 °C in a third and final annealing step (Figure 1d).

In the absence of phenyl side groups, the lateral coupling of the corresponding 7-13-AGNRs gives rise to a NPG structure with two equivalent interribbon coupling configurations.^21^ However, the side groups of *Ph*-7-13-AGNR increase the coupling degrees of freedom, giving rise to the three nonequivalent configurations depicted in Figure 1d. The phenylated GNRs can couple from the *para* and *meta* sites, giving rise to *para–para* (*pp*), *para–meta* (*pm*), and *meta–meta* (*mm*) phenylene bridges. The *ortho* cross-coupling configuration is sterically hindered by the hydrogens at the adjacent phenyl unit and the fact that it implies the formation of more than one bond.

Interestingly, chiral interactions seem to play a fundamental role in determining the relative abundance of each coupling configuration. From pure energetic arguments and based on the coupling energies obtained for free-standing NPGs, the *pm* configuration is the most stable, followed by *mm* and *pp*, which are 140 and 510 meV less stable, respectively (see Section 1.4 of the Supporting Information for details of the calculation). However, although we do observe a preference of the *mm* over the *pp* configuration, we only find a marginal number of the most stable *pm* configuration. This can be explained by noting that this configuration is the only one that requires a heterochiral pair of ribbons (Figure 1d), something that is hindered by the chiral phase separation undergone during the polymerization step. The scenario of the chiral hindering of the *pm* coupling is supported by the fact that the few *pm* coupled ribbons are always found at the periphery of the NPG sheets, the only site where a ribbon of opposite chirality can join a homochiral cluster. Regarding the *pp* and *mm* bridge configurations, these often appear mixed within a NPG sheet, but one can also find single configuration ensembles as the one shown in Figure 1d (center). The *mm* bridge is the most abundant, in agreement with the differences in the formation energy of the two.

**Figure 2:**
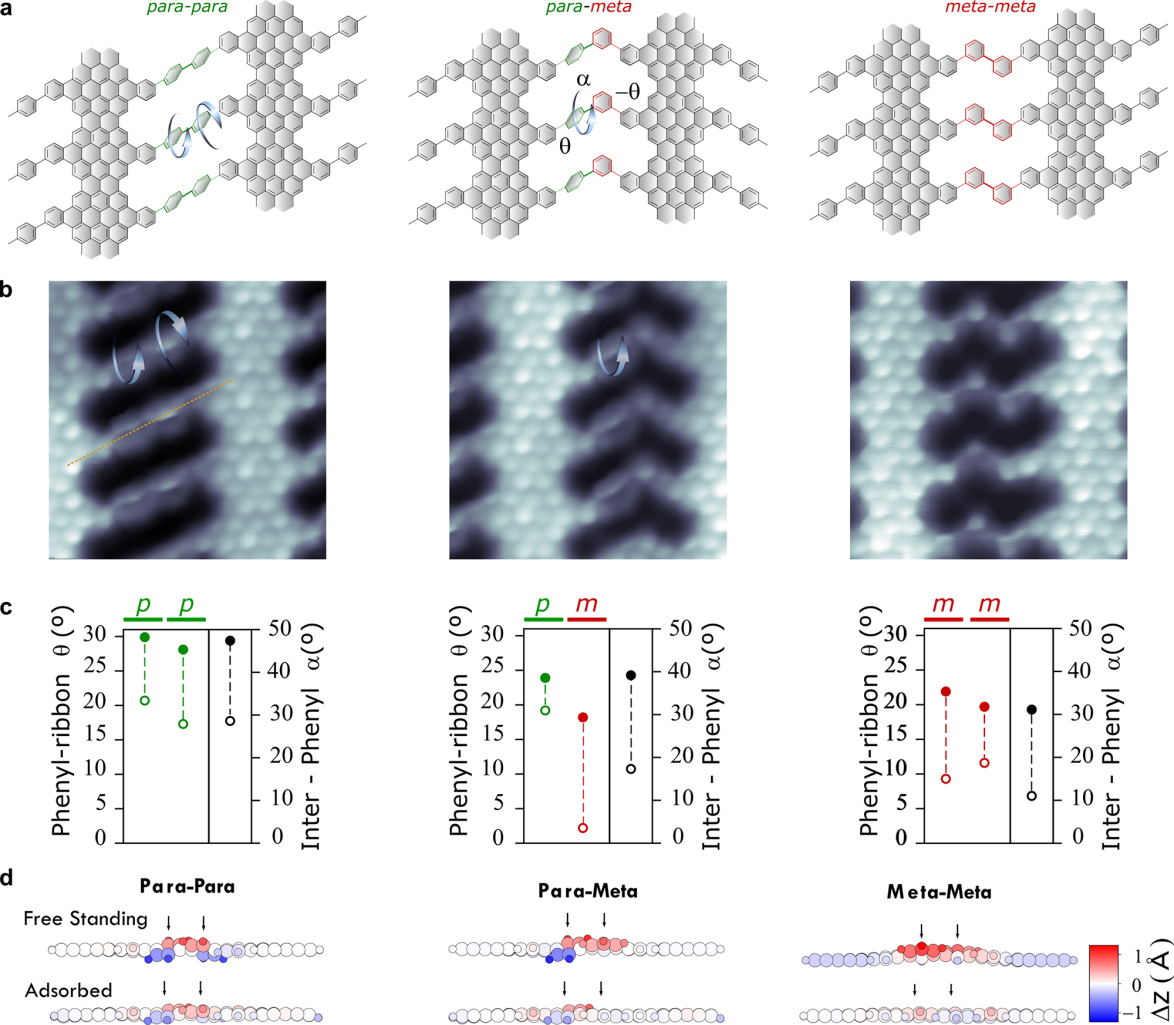
Phenylene twist at NPG bridges. (a) Schematics of the three type of bridge configurations, with the selective twist found by STM for the *para*-phenylenes indicated by arrows. (b) BR-STM close-up images of the corresponding bridge configurations. The sharp features observed at *para*-phenylene sites are attributed to the out-of-plane tilt induced by the twist. In the *pp* bridge, the two phenyl units are tilted toward opposite directions with respect to the *para* bond axis (dashed yellow line). (c) Phenyl-ribbon twist angle θ (color circles) and interphenyl twist angle α (black circles), obtained by DFT for freestanding (solid circles) and Au-supported (open circles) NPGs. Angles are defined in (a) (see text for further description). (d) Side views of the corresponding atomic structures, where the interphenyl twist and the local distortion of the backbone can be appreciated by the out-of-plane distortion indicated in the red/blue color scale.

Looking into the bridge conformation in more detail by using BR-STM images as the ones shown in Figure 2b, one can see that the appearance of the bridging phenylenes depends on the bonding configuration. *p*-Phenylenes appear as sharp contrast features instead of hexagonal rings, a common characteristic of nonplanar molecular groups that interact with the CO-functionalized tip used for BR-STM imaging.^29^ In pure *pp* bridges, the clockwise/anticlockwise conformation of the two phenyl units with respect to the *para* bond axis reveals the interphenyl twist. The appearance of *m*-phenylenes, instead, resembles that of the planar rings of the backbone. This selective twist of *p*-phenylenes, also observed in coupled pairs of phenylated chevron type GNRs,^30^ cannot be solely explained by steric hindrance arguments, since this affects similarly both *p* and *m* configurations, as it is well-known for polyphenylene chains.^31^ Indeed, DFT calculations performed for the free-standing NPG structures reveal a significant twist for the three type of isomers, as shown in Figure 2c. For this analysis, we define two different twist angles, the first defined by planes of the outermost benzene ring of the GNR backbone and the adjacent phenyl (θ) and the second between the two phenylenes at the bridge (α). Note that α = 2 × θ only when the outermost benzene ring remains coplanar to the backbone, something that will not be the case due to small nonplanar distortions induced at the backbone to accommodate steric repulsion, as shown later. As reference, if we compare the interphenyl twist angle α = 47° obtained for the *pp*-NPG with values found in the literature for poly-*p*-phenylene, we see that it lies within the α = 40° and 70° calculated for infinite^31^ and finite^32^ chains, respectively. When comparing the twist angles of the free-standing NPG (solid circles) to calculations carried out including the Au(111) substrate (dashed circles), we see an overall planarization of the structure, a clear effect of the interaction with the substrate that competes with steric hindrance. The effect is, however, much more pronounced for the *m*-phenylenes, as found in the experimental data. The differences are most visible in the mixed *pm* bridge, where the *m*-phenylene turns nearly coplanar to the backbone by the interaction with the underlying substrate. In the *mm* bridges, phenyls still have to maintain a minimum tilt in order to avoid each other, but the twist angles are considerably smaller than those of the *pp* bridge. In order to account for the different degree of planarization, one has to pay attention to the distortion of the backbone structure induced by the twisting. The side views of the relaxed structures depicted in Figure 2d contain the key for such differences. Whereas *p*-phenylenes do not significantly distort the backbone structure, *m*-phenylenes induce a substantial out-of-plane distortion at the peripheral units (see Figure 2d for distortion maps). The substrate, in addition to tending to planarize both *p* and *m* bridge phenylenes similarly by the local π interaction, also counteracts the backbone distortion necessary for the twist of the latter, consequently planarizing *m*-phenylenes more effectively.

A qualitative assessment of the capability of the substrate interaction to tune the twist angle can be made by comparing the BR-STM image of the *pp* bridged NPG with that of the isolated ribbon. We can see that the interaction with the substrate can easily surpass the steric hindrance of the single neighbor found in the ribbon, as the phenylene appears to be coplanar to the backbone in this case (Figure 1c right). This observation is corroborated by our DFT analysis of the isolated *Ph*-7-13-AGNR (see Figure S8). It is only when we add a second neighbor in the *pp* bridges that the steric repulsion becomes strong enough to maintain a phenylene twist even in the presence of the substrate (Figure 2b left).

**Figure 3:**
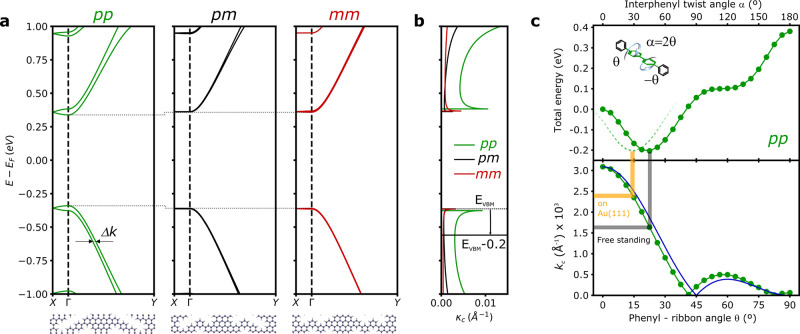
Electronic properties of phenylene-bridged NPG. (a) Electronic band structures of free-standing *pp*-, *pm*-, and *mm*-NPG. Γ*Y* and Γ*X* correspond to the longitudinal (i.e., along the ribbon) and transversal directions of the 2D nanostructures. To account solely for the effect of the chemical bond, the structures are forced to remain coplanar in the relaxation. Atomistic models of the corresponding unit cells are displayed below. (b) Interchannel coupling coefficient κ_c_ = Δ*k*/4 obtained from the momentum difference of the frontier bands. (c) Evolution of total energy relative to the coplanar configuration (top) and interchannel coupling coefficient κ_c_ as a function of interphenyl twist angle for *pp*-NPG, measured at *E* – *E*_VBM_= −0.2 eV (horizontal line in (b)). The catafused benzene of the backbone is forced to be coplanar with the rest of the backbone, so that α = 2θ. The *k*_c_ values corresponding to the twist angles in the relaxed free-standing and Au supported structures are indicated with gray and yellow lines.

The conformational flexibility provided by the phenylene bridges are expected to affect the interribbon electronic coupling and hence the quantum electron transport across the NPG sheets and its anisotropy. We explore that by analyzing the band structure of the different configurations and, in the case of *pp*-NPG, its evolution with the twist angle. Figure 3a compares the frontier band structure of free-standing NPGs for the three coupling configurations. In this case, the structures have been relaxed by imposing coplanarity in order to account solely for the effect of chemical bonding configuration. The most notable differences are found between *pp*-NPG and the other two configurations containing at least one *m*-phenyl at the bridge. One can see that for *pp*-NPG the degenerated valence (VB) and conduction (CB) bands split, leading to a slight reduction of the band gap and a finite dispersion in the transversal direction (Γ*X*). All these are manifestations of a finite electron transmission across the phenylene bridges. In contrast, adding one *m*-phenyl to the bridge seems to switch off the interribbon coupling effectively, quenching the band splitting and the transversal band dispersion. The fact that a single *m*-phenyl is enough to effectively quench transmission across the bridge is in agreement with calculations reporting that a single *meta* kink is enough to effectively disrupt electron delocalization across polyphenylene zigzag chains.^32^ We attribute this quenching of transmission to the destructive interferences produced in the transport across *m*-phenylenes, as has been corroborated by previous electron propagation simulations on theoreticaly proposed single-phenyl bridged NPG structures.^25^ Similar interference effects have also been found in experimental^33−35^ and theoretical^31,36,37^ studies of transport across single-molecule junctions with broken or cross conjugation.

A more quantitative analysis of the interribbon coupling can be obtained by measuring the momentum splitting between the otherwise degenerated VB and VB-1 bands (a similar analysis could be carried out with CB and CB+1). The momentum splitting Δ*k* directly relates to the interchannel coupling coefficient κ_c_ that mixes the leaking wave functions of adjacent ribbons as *k*_c_ = Δ*k*/4.^24^ The resulting energy-dependent *k*_c_(*E*) of the frontier bands is plotted for all configurations in Figure 3b. As reference, we see that, at 0.2 eV below the VB maximum (VBM) (black line in Figure 3b), κ_c_ is reduced by more than a factor of 5 when we introduce a *m*-phenyl in the bridge.

In contrast to the abrupt on/off switching of the interribbon coupling provided by this robust chemical knob, the conformational degree of freedom rendered by the interphenyl twist angle can enable a more gradual knob. We analyze this by tracking the evolution of κ_c_ in *pp*-NPG as a function of twist angle. For the calculation, we consider the energy 0.2 eV below the VBM (black line in Figure 3b), and a rigid backbone, so that α = 2θ. The result is plotted in Figure 3c together with the total energy variations induced by the phenyl twist. One can see that the maximum coupling is for the coplanar system where π overlap is maximized and decays to zero for the orthogonal bridge configuration (α = 2θ = 90°). Beyond this angle, we find a second minima at α = 2θ = 180° where, even if the bridge phenyls are coplanar, conjugation is now broken by their orthogonal configuration with respect to the backbone (i.e., θ = 90°). The small maximum between the two nodes never reaches the absolute maxima of the coplanar configuration. This trend is perfectly reproduced by a model that considers uniquely the π overlap across the bridge with κ_c_ = κ_c_(0°) × cos^2^(θ) × cos(2θ) (blue line in Figure 3c bottom).

From the angular evolution of κ_c_ described above, α = 0–90° is clearly the region of interest for tuning interribbon coupling. Remarkably, the relaxed interphenyl twist angle lies exactly at the center of this region for the free-standing structure, as given by the total energy minimum (Figure 3c top). The capability to tune the interribbon coupling around this value is demonstrated by noting that the reduction of twist angle from α = 47° to α = 29° found for Au supported NPG results in an increase of κ_c_ by 44% (thick gray and yellow lines in Figure 3c bottom). The angle-dependent energy calculations also provide an estimation of the thermally induced oscillations of the interphenyl twist angle. Whereas thermal effects are negligible at the temperature of the STM experiments (5 K), at room temperature, the rotational energy of 1/2*k*_B_*T* = 12.7 meV results in an oscillation of ±8° around the minimum energy value for the free-standing case. When supported on Au, these values are expected to be even smaller.

**Figure 4:**
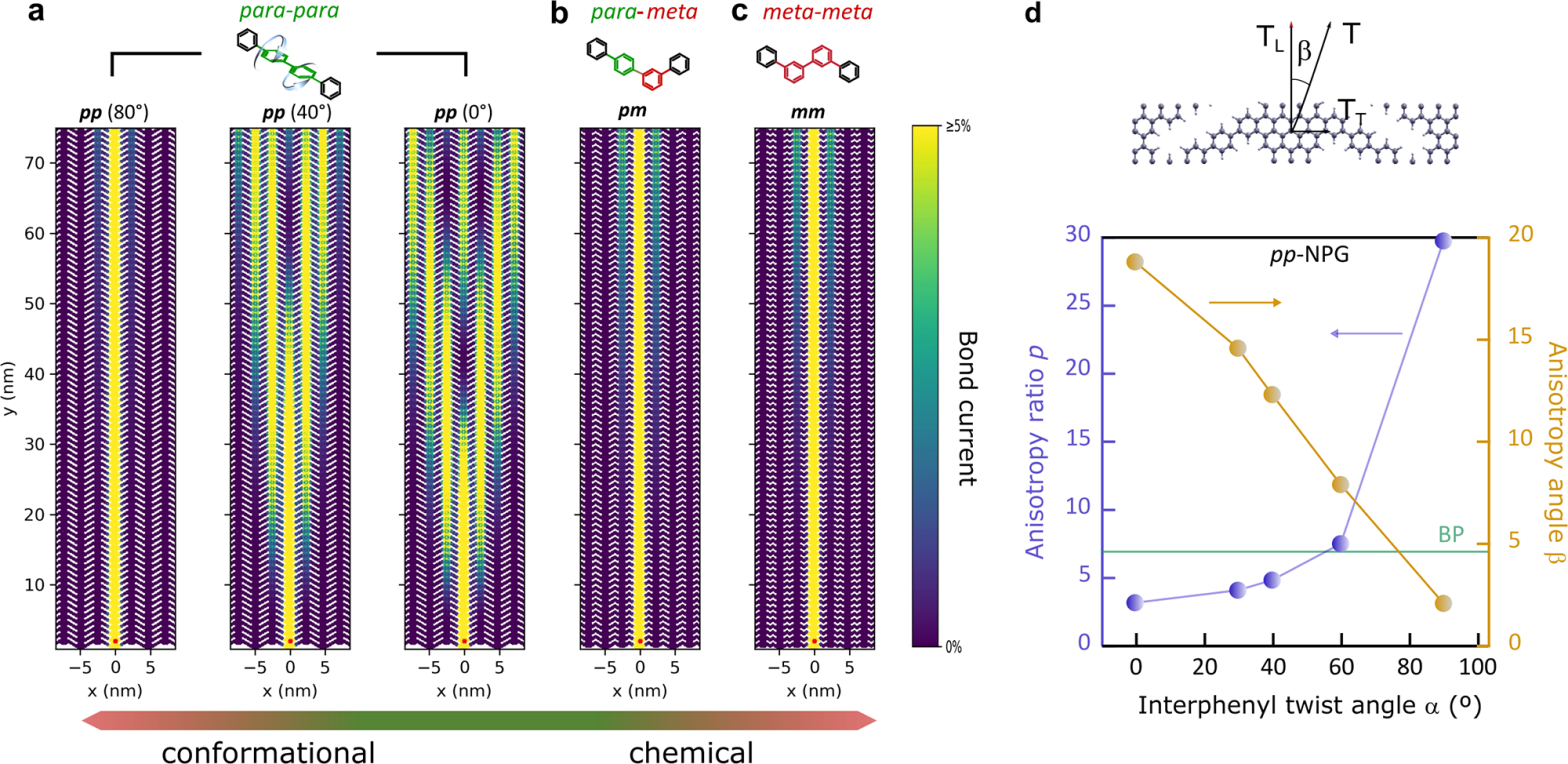
Bridge engineering of current injection. (a–c) Bond current maps in large-scale NPG fragments comprising seven 75 nm long graphene nanoribbons. The maps show the propagation of currents injected at *E* – *E*_VBM_ = −0.2 eV at the lower end of the central ribbon (red dot) for different interphenyl twist angles of *pp*-NPG (a) and coplanar *pm*-NPG (b) and *mm*-NPG (c). The color bar is saturated above 5% of the maximum value in order to detect the interference patterns produced by the current transmitted across the ribbons. (d) Evolution of the anisotropy ratio *p* = tan(90 – β) = *T*_L_/*T*_T_ with twist angle for *pp*-NPG, where *T*_L_ and *T*_T_ are the longitudinal and transversal transmissions, respectively. The horizontal green line corresponds to the effective mass ratio calculated for black phosphorus.^38^

The tunability of interribbon coupling and its effect on electron transport can be directly visualized by carrying out quantum simulations of the propagation of electron current within the NPG. Figure 4a–c summarizes the results obtained for each configuration and for different twist angles in the case of *pp*-NPG. The color maps displayed here correspond to bond currents; i.e., the electron flow across the *p*_*z*_ orbitals of each carbon atom when electrons are injected from a metallic tip in contact with one of the ribbons of the NPG fragment at the bottom end (red dot) and propagate toward a drain electrode that connects all ribbons at the top end (see Methods in the Supporting Information for more details). As in the analysis of Figure 3, coplanar *pp* (Figure 4a right), *pm* (Figure 4b), and *mm* (Figure 4c) configurations have been compared first to disentangle the effects of the two degrees of freedom. The difference between the former and the ones containing at least one *m*-phenyl at the bridge is evident. In *pp*-NPG, the injected current spreads across adjacent ribbons, forming the so-called Talbot interference patterns.^24^ In contrast, in the *m*-phenyl containing NPGs, the current is basically confined within a single ribbon, similar to that found for other *meta*-bridged NPG structures.^25^ The interesting situation comes when we turn the conformational degree of freedom in the *pp*-NPG. Here, we see how the divergence of the Talbot interference pattern is gradually reduced as we increase the interphenyl twist angle and completely fades out close to α = 90°, where π orbitals at the bisphenyl bridge turn orthogonal. These modulations of the quantum interferences mimic the continuous evolution of κ_c_ found for varying interphenyl twist angle.

The quantum interferences obtained for the longitudinal propagation of injected point charges are indicative of a strong anisotropy in the electronic transport, and thus, the interphenyl twist can also be seen as a tool for tailoring the overall electronic anisotropy in this nanomaterial. This can be measured by computing the longitudinal (*T*_L_) and transversal (*T*_T_) transmissions by using orthogonal pairs of semi-infinite electrodes corresponding to the pristine, orthogonal unit cell (Figure 4d top) repeated along the transverse and longitudinal ribbon directions, respectively. From the 2D transmission vector *T⃗* = *T⃗*_L_ + *T⃗*_T_, one can obtain the anisotropy angle β (see sketch in Figure 4d), and the corresponding anisotropy factor *p* = tan(90 – β) = *T*_L_/*T*_T_. The computed values of *p* and β are represented in Figure 4d as a function of interphenyl twist angle. One can see that the bridge conformation can modulate the anisotropy of the material by an order of magnitude. The whole modulation window is within the strong anisotropy regime, well above any experimental value reported for a 2D material (*p* < 3).^39−43^ The computed transmission ratio can be directly compared to effective mass ratios calculated for other 2D materials, which are equivalent within the parabolic band approximation. The values obtained for a model anisotropic 2D material such as black phosphorus^38^ lie in the lower branch of the modulation window of the *pp*-NPG (see horizontal line).

## Conclusions

In conclusion, with the synthesis of atomically precise nanoporous graphene structures consisting of graphene nanoribbons connected by flexible phenylene bridges, we open a way of tailoring quantum transport and the anisotropy in 2D materials. Our calculations demonstrate that, in contrast to the effective interribbon decoupling offered by *meta* phenyls, interesting to confer a robust, absolute 1D anisotropy to the nanomaterial, *para* bridges can be designed to tune this anisotropy continuously in a wide range by varying the interphenyl twist angle. This could be modulated by external stimuli such as strain or electric fields applied to functionalized polar phenylene bridges or more statically by using substrates with different degrees of interactions. We foresee that this molecular bridge strategy could also be used to tailor the phononic anisotropy, leading to novel approaches in the search of thermoelectric nanomaterials.

## Supporting Information Available

Experimental and theoretical methods, analysis on chiral polymer chain assembly, electronic properties of *Ph*-7-13-AGNR, and phenyl planarization of individual ribbons (PDF)
