# Supplementary material for: Molecular Bridge Engineering for Tuning Quantum Electronic Transport and Anisotropy in Nanoporous Graphene
Source: J Am Chem Soc. 2023 Mar 29;145(16):8988–95. doi: 10.1021/jacs.3c00173 (PMC10141406; doi:10.1021/jacs.3c00173)
Supplement: Supplementary file 1 — ja3c00173_si_001.pdf [file ja3c00173_si_001.pdf]

# Supporting Information for Molecular bridge engineering for tuning quantum electronic transport and anisotropy in nanoporous graphene

César Moreno,[*,†,‡] Xabier Diaz de Cerio,[¶] Manuel Vilas-Varela,[§] Maria Tenorio,[‡,△] Ane Sarasola,[¶,∥] Mads Brandbyge,[⊥] Diego Peña,[*,§] Aran Garcia-Lekue,[*,¶,#] and Aitor Mugarza[*,‡,@]

†*Departamento de Ciencias de la Tierra y Fisica de la Materia Condensada, Universidad de Cantabria, 39005 Santander, Spain*

‡*Catalan Institute of Nanoscience and Nanotechnology (ICN2), CSIC and The Barcelona Institute of Science and Technology, Campus UAB, Bellaterra, 08193 Barcelona, Spain*

¶*Donostia International Physics Center, Paseo Manuel de Lardizabal 4, 20018 San Sebastian, Spain*

§*Centro de Investigación en Química Biolóxica e Materiais Moleculares (CiQUS) and Departamento de Química Orgánica, Universidade de Santiago de Compostela, 15782 Santiago de Compostela, Spain*

∥*Departamento de Física Aplicada, Universidad del País Vasco/Euskal Herriko Unibertsitatea (UPV/EHU), 20018 Donostia, Spain*

⊥*Department of Physics, Technical University of Denmark, DK-2800 Kongens Lyngby, Denmark*

#*Ikerbasque, Basque Foundation for Science, 48013 Bilbao, Spain*

@*ICREA – Institució Catalana de Recerca i Estudis Avançats, Lluis Companys 23, 08010 Barcelona, Spain*

△*Current address: IMDEA Nanoscience, Cantoblanco, Madrid, Spain*

E-mail: cesar.moreno@unican.es; diego.pena@usc.es; wmbgalea@ehu.eus; aitor.mugarza@icn2.cat

# 1 Methods

## 1.1 Synthesis of the molecular precursors

*General methods:* Starting materials were purchased from TCI or Sigma-Aldrich and used without further purification. Reactions were carried out in flame-dried glassware and under argon using Schlenk techniques. Thin-layer chromatography (TLC) was performed on Silica Gel 60 F-254 plates (Merck). Column chromatography was performed on silica gel (40-60 $\mu$m). NMR spectra were recorded on a Bruker Varian Mercury 300 spectrometer. 2,2'-Dibromo-9,9'-bianthracene (**1**) was synthesized following a reported procedure.[1] DBP-DBBA was obtained in two steps from compound **1** following the procedure shown in Fig. S1.

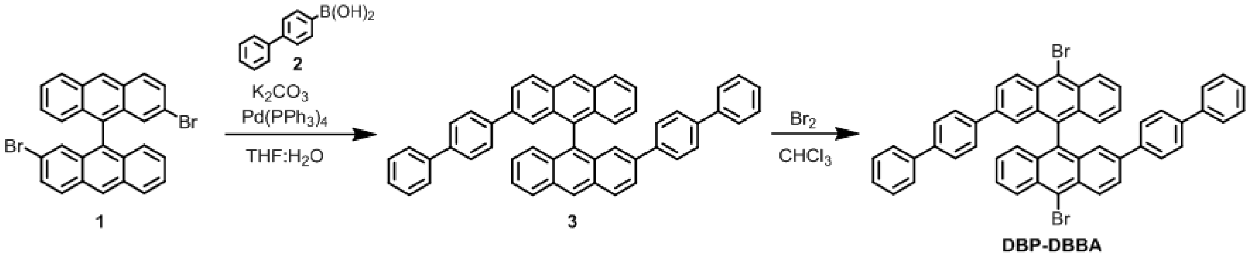


Fig. S1: Synthesis of DBP-DBBA.

*Synthesis of 2,2'-di([1,1'-biphenyl]-4-yl)-9,9'-bianthracene (**3**):* The complex $Pd(PPh_3)_4$ (12 mg, 0.01 mmol) was added over a deoxygenated mixture of 2,2'-dibromo-9,9'-bisantracene (**1**, 50 mg, 0.10 mmol), boronic acid **2** (97 mg, 0.49 mmol) and $K_2CO_3$ (203 mg, 1.47 mmol) in THF:$H_2O$ (1:1, 10 mL). The resulting mixture was heated at reflux for 20 h. After cooling to room temperature, phases were separated, and the aqueous layer was extracted with $Et_2O$ (3 x 10 mL). The combined organic phases were dried over anhydrous $Na_2SO_4$, filtered and evaporated under reduced pressure. The crude product was purified by column chromatography ($SiO_2$; hexane:$CH_2Cl_2$ 4:1 to 3:1) affording compound **3** (63 mg, 98%) as a yellow solid (m.p. 308°C). $^1$**H-NMR** (300 MHz, $CDCl_3$) $\delta$: 8.72 (s, 2H), 8.27 (d, $J$ = 8.8 Hz, 2H), 8.17 (d, $J$ = 8.5 Hz, 2H), 7.79 (dd, $J$ = 8.8, 1.8 Hz, 2H), 7.52 – 7.44 (m, 10H), 7.42 – 7.29 (m, 12H), 7.20 – 7.10 (m, 4H) ppm. $^{13}$**C-NMR-DEPT** (75 MHz, CDCl3) $\delta$:

140.5 (2C), 140.0 (2C), 139.9 (2C), 137.8 (2C), 133.3 (2C), 132.1 (2C), 131.9 (2C), 131.7 (2C), 130.8 (2C), 129.3 (2CH), 128.7 (5CH), 128.6 (2CH), 127.6 (5CH), 127.3 (7CH), 126.9 (5CH), 126.0 (2CH), 125.5 (2CH), 125.4 (2CH), 124.2 (2CH) ppm. See Fig. S2 for NMR data. **MS (EI)** m/z (%): 658 (M+, 100), 329 (31). **HRMS**: $C_{52}H_{34}$; calculated: 658.2661, found: 658.2643.

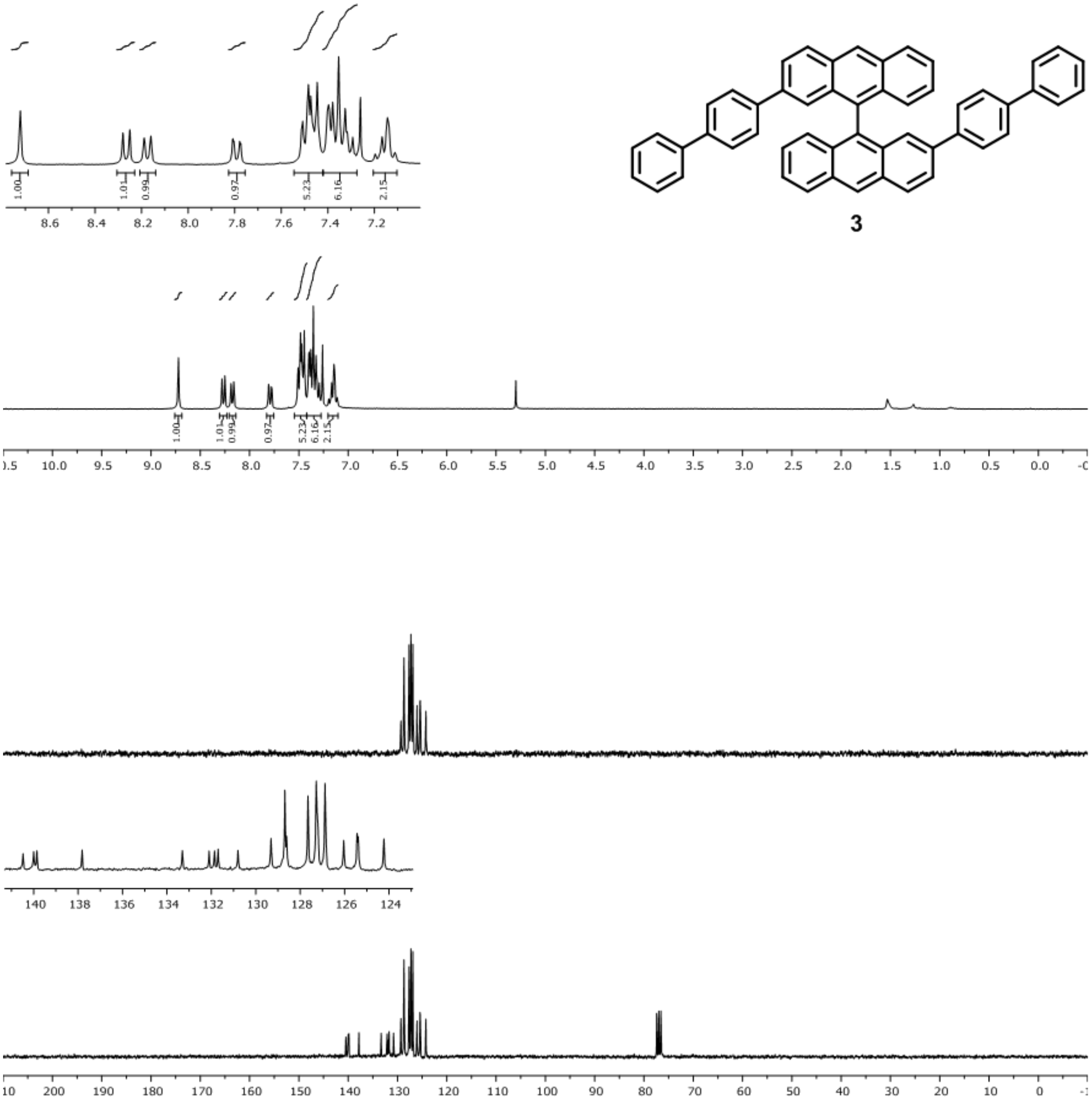


Fig. S2: $^{1}$H and $^{13}$C-NMR spectra of compound **3**.

*Synthesis of 2,2'-di([1,1'-biphenyl]-4-yl)-10,10'-dibromo-9,9'-bianthracene (DBP-DBBA):* A solution of $Br_2$ (0.2 M in $CHCl_3$, 668 $\mu$L, 0.13 mmol) was dropwise added over a solution of bianthracene **3** (40 mg, 0.06 mmol) in $CHCl_3$ (5 mL) at 0°C. Then, the mixture was allowed to reach room temperature and stirred for 16 h. Aqueous solution of $Na_2S_2O_3$ (10%, 5 mL) was added and phases were separated. The organic phase was dried over anhydrous $Na_2SO_4$, filtered and evaporated under reduced pressure. The crude product was purified by column chromatography ($SiO_2$; hexane:$CH_2Cl_2$ 4:1 to 2:1) affording DBP-DBBA (42 mg, 85%) as a yellow solid (m.p. 334°C). $^1$**H-NMR** (300 MHz, $CDCl_3$) $\delta$: 8.82 (d, $J$ = 9.1 Hz, 2H), 8.72 (d, J = 8.9 Hz, 2H), 7.92 (d, $J$ = 9.2 Hz, 2H), 7.59 (t, $J$ = 7.7 Hz, 2H), 7.49 (m, 6H), 7.41 – 7.28 (m, 10H), 7.28 – 7.24 (m, 4H), 7.26 – 7.14 (m, 2H), 7.10 (d, $J$ = 8.8 Hz, 2H) ppm. $^{13}$**C-NMR-DEPT** (75 MHz, $CDCl_3$) $\delta$: 140.5 (2C), 140.3 (2C), 138.9 (2C), 138.4 (2C), 133.3 (2C), 132.7 (2C), 132.5 (2C), 130.5 (2C), 129.7 (2C), 129.0 (2CH), 128.7 (4CH), 128.2 (2CH), 127.7 (6CH), 127.4 (4CH), 127.3 (4CH), 127.1 (2CH), 126.9 (4CH), 126.6 (2CH), 124.2 (2CH), 124.0 (2C) ppm. See Fig. S3 for NMR data. **MS (EI)** m/z (%): 816 (M+, 100), 736 (9), 658 (10), 408 (17). **HRMS**: $C_{52}H_{32}Br_2$; calculated: 814.0871, found: 814.0876.

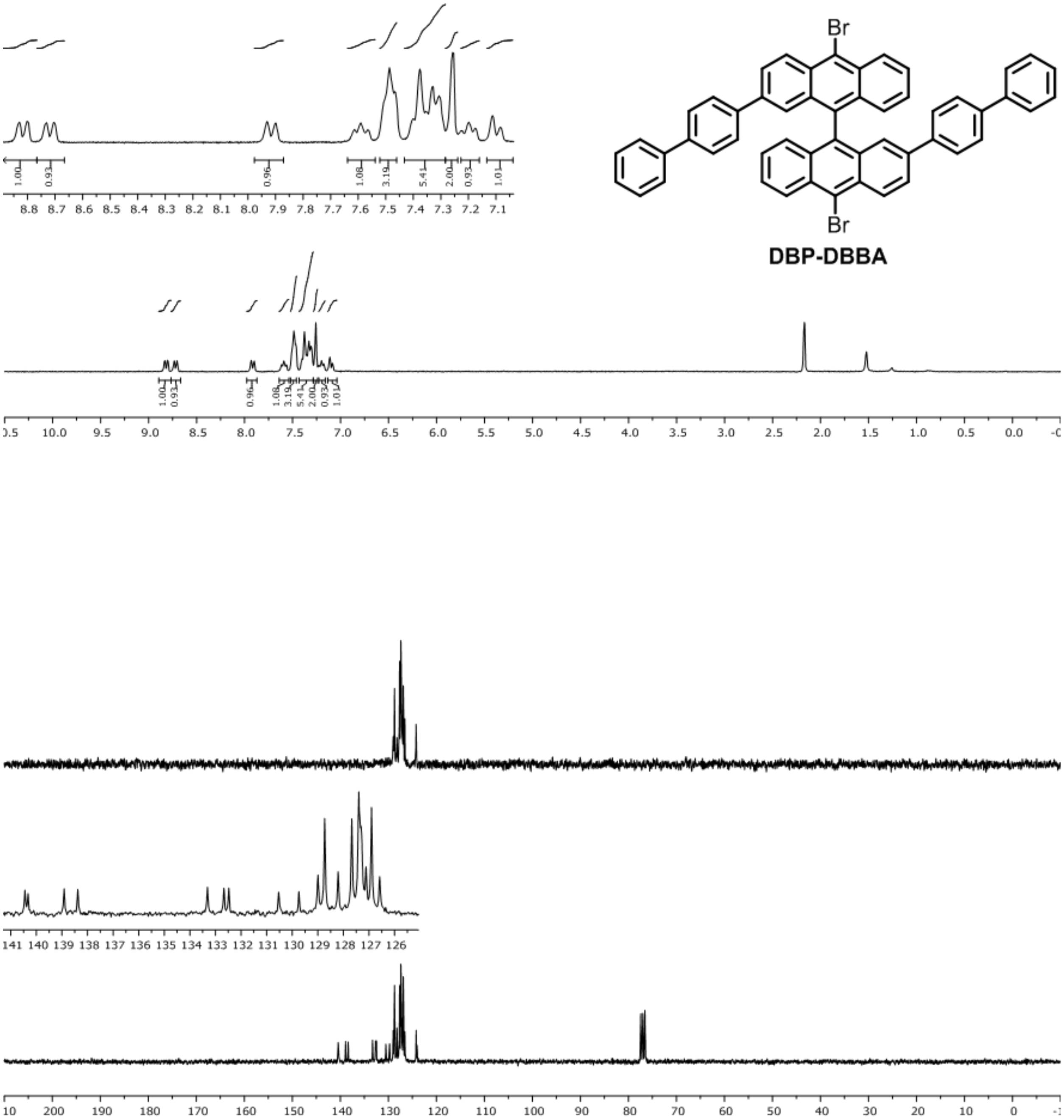


Fig. S3: $^{1}$H and $^{13}$C-NMR spectra of DBP-DBBA.

## 1.2 Sample preparation and on-surface synthesis

The Au(111) single crystal was prepared by repeated sputter-anneal cycles using Ar+ ions at an energy of 1 keV and annealing to 470°C. Precursors were sublimated from a commercial Dodecon OMBE four-fold Knudsen cell with the crucible at 355°C and the Au(111) held at room temperature. Sample temperature was measured by using a thermocouple directly in contact to the sample. The base pressure during evaporation was below $1 \times 10^{-9}$ mbar.

## 1.3 Experimental details on the imaging method

Bond-resolved STM images were acquired by functionalizing the tip with a CO molecule. For that, the molecules were first introduced on the Au surface by dosing the cryostat with CO gas at a pressure of $5 \times 10^{-7}$ mbar for 30 seconds in a temperature range from 5 to 15 K. The CO was then picked up by the tip by applying pulses of 2.5 V at random positions or by gentle tip indentations on the Au surface until the resolution was suddenly improved. The higher resolution of these tips are related to dynamic effects in the CO-metallic tip junction as it interacts with the atoms beneath.

## 1.4 *Ab-initio* Calculations and quantum electron transport simulations

The atomic and electronic structure calculations were performed using DFT as implemented in the SIESTA code.[2,3] To explore the conformational details of phenylene twist at NPG bridges (Fig. 2), and in order to reduce the computational cost, we carried out free-standing and surface supported calculations for a coupled pair of ribbons, where the peripheral phenyls were not included. We used a supercell description of the system, made up of a slab containing 4 layers of a Au(111) 7×6 unit cell with a double ribbon on top of one of the Au surfaces. For all relaxations, the outermost part of the double ribbons were maintained fixed so as to mimic the nanoribbon clustering observed experimentally. For the Au supported

relaxations, the top most layer of the substrate was also relaxed. In all cases, forces were less than 0.01 eV/Å. The energy cut-off was fixed at 300 Ry and a 1×5×1 $k$-mesh was used, where 5 $k$-points were taken in the direction of the ribbon.

DFT-based total energy calculations for the free-standing systems allowed us to estimate the relative stability of the various bridge configurations. We find that $pm$ is the energetically most favourable conformation (lowest total energy), while the $mm$ and $pp$ have 140 and 510 meV higher total energies, respectively.

For the free-standing calculations of the different phenylene-bridged NPGs (Fig. 3), a 4.751 nm × 0.866 nm supercell was employed, containing 104 carbon atoms and 36 hydrogen atoms. A vacuum region of 20 Å was introduced perpendicular to the surface in order to avoid non-physical interactions between periodic images. Core electrons were represented by norm-conserving Troullier-Martins pseudopotentials,[4] while a linear combination of atomic orbitals (LCAO) was used as a basis set for valence electrons. In particular, a double-$\zeta$ polarized (DZP) basis set with a 0.01 Ry energy shift[5] was selected. Exchange-correlation was treated by GGA-PBE.[6] The Brillouin zone was sampled by a 4×15×1 Monkhorst-Pack $k$-mesh[7] and a mesh-cutoff of 400 Ry was employed to define the real-space grid. Atomic coordinates were optimized until forces between atoms were lower than 0.01 eV/Å and the atomic structure was constrained to remain planar during relaxation. Cell relaxation was performed with a pressure threshold of 0.25 GPa. For the simulations of tilted $pp$-NPG, atomic structures were built starting from the optimized planar $pp$-NPG and manually rotating the phenyl rings in the bridges. No further relaxation was performed in these geometries.

Quantum electron transport simulations were carried out using the TRANSIESTA utility TBTRANS,[8] which combines tight-binding (TB) Hamiltonians and Non-equilibrium Green's Functions (NEGF) to compute transport. Accurate "pruned" DFT hamiltonians were constructed using SISL[9] into a reduced set of atomic orbitals: $s$, $p_x$, $p_z$ and $p_y$ for carbon atoms sitting in the bridges, and $p_z$, $Pd_{xz}$ and $Pd_{yz}$ for the rest of carbon atoms (respectively blue and grey atoms in Figs. S4 and S5). $Pd_{xz}$ and $Pd_{yz}$ refer to polarization (P) orbitals.[10] This

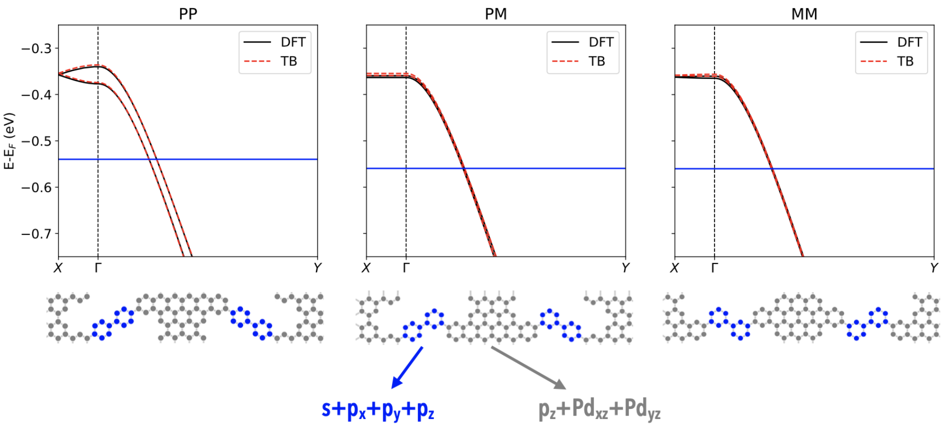


Fig. S4: Band structure comparison of DFT (black solid lines) versus DFT-parametrized TB (red dashed lines) in the occupied low energy region of planar *pp* (left), *pm* (middle) and *mm*-NPG (right). The horizontal blue solid line indicates the energy at which charge carriers are injected in the large-scale transport simulations, -0.2 eV away from the valence band maxima (VBM) of each case ($E_i = VBM - 0.2$ eV).

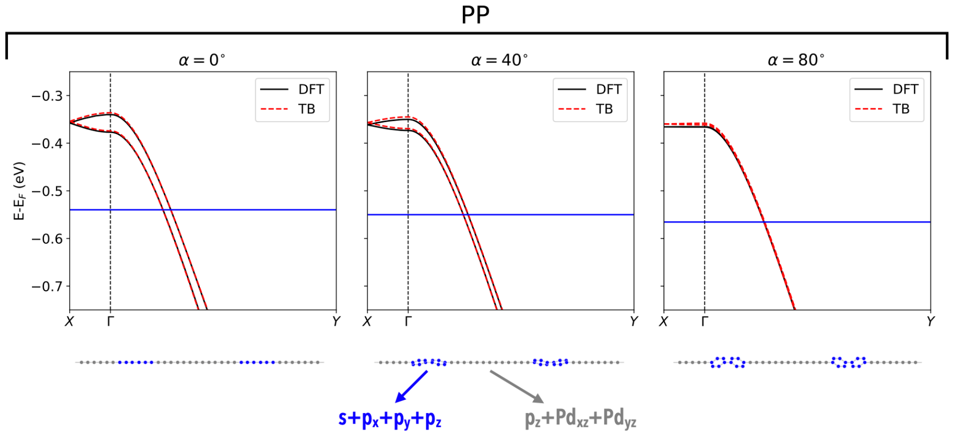


Fig. S5: Band structure comparison of DFT (black solid lines) versus DFT-parametrized TB (red dashed lines) in the occupied low energy region of tilted *pp*-NPGs: $\alpha = 0°$ (left), $\alpha = 40°$ (middle) and $\alpha = 80°$ (right). The horizontal blue solid line indicates the energy at which charge carriers are injected in the large-scale transport simulations, -0.2 eV away from the valence band maxima (VBM) of each case ($E_i = VBM - 0.2$ eV).

multi-scale approach[10,11] allows treating current point-injection into systems up to ~100.000 atoms. In particular, 42.759 nm × 64.950 nm devices were considered here (9 × 75 NPG cells), consisting of 70.200 atoms. Self-energies and complex absorbing potentials are used

to mimic infinite systems and avoid spurious reflection of the current at the edges of the device region.

## 2 Analysis of interpolymer distance

The interpolymer configuration has been analyzed by studying large and small scale STM images of the polymer chain ensembles. The average interchain distance, which varied in the range of d = 2.0-2.2 nm, has been obtained from Fast Fourier Transforms of the images. Smaller scale images, on the other hand, have been used to study the relative conformation, by tracking the relative position of the lobes of adjacent polymers. From this analysis one can also obtain the interlobe distance within the chains, which is of a = 0.75 nm, in line what that reported for staggered anthracene polymers.[12] By using these distances to represent the the homochiral and heterochiral assembly configurations, represented in Fig. S6c, it can be concluded that the homochiral one is more favourable.

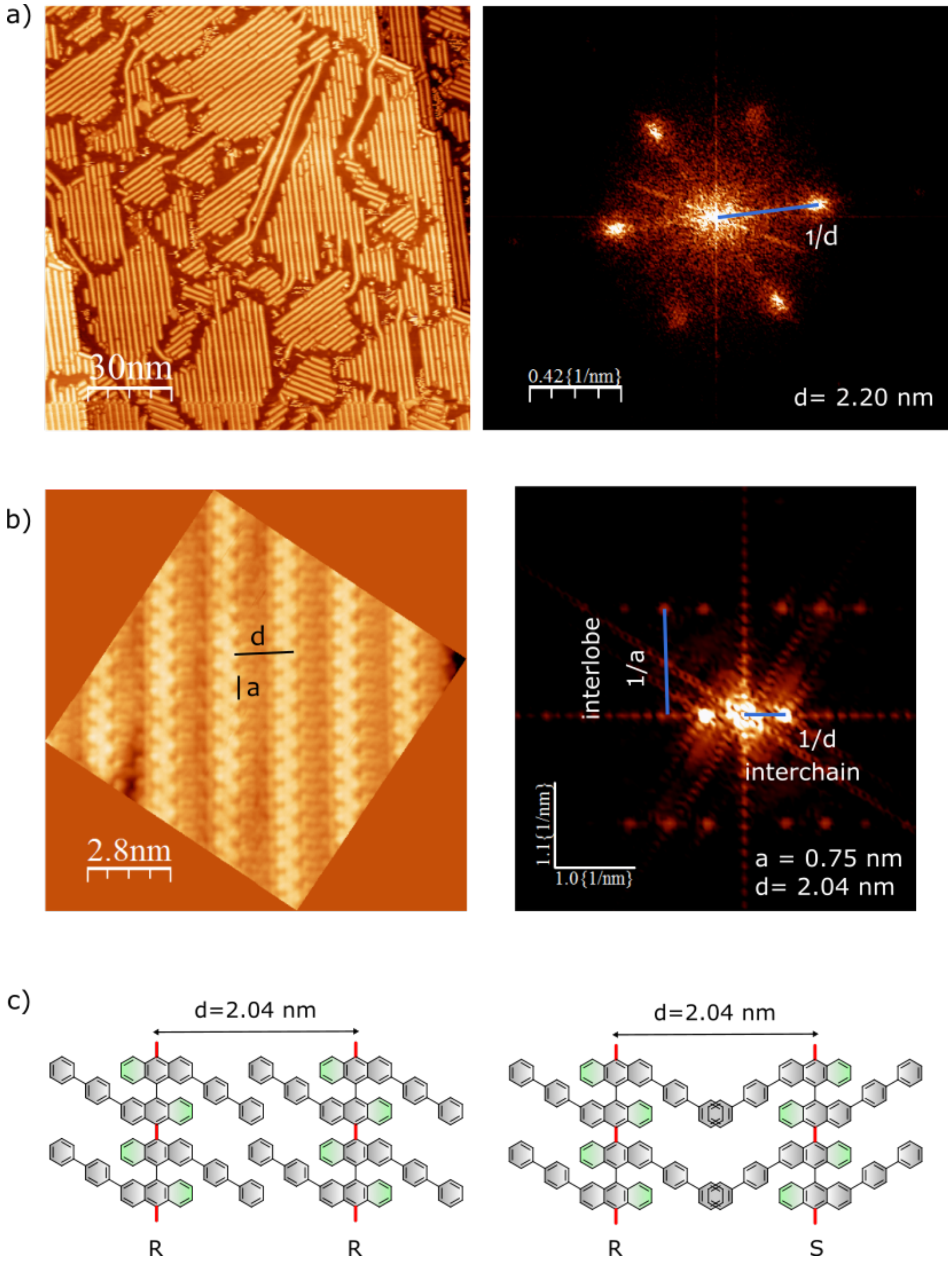


Fig. S6: Large (**a**) and small (**b**) scale STM topographic images of the self-assembled arrays of linear polymer chains obtained after the Ullmann coupling reaction induced at step $T_1 = 200°C$, also shown in Fig. 2 of the main manuscript. The corresponding FFT images on the right are used to obtain the interpolymer distance, as well as the interlobe distance of a = 0.75 nm within polymers. **c**, Schematic representation of chiral and racemic dimers at the interlobe distance obtained in **b**, showing how the chiral pair results in a more favourable interdigitated configuration.

# 3 Electronic properties of *Ph*-7-13-AGNR

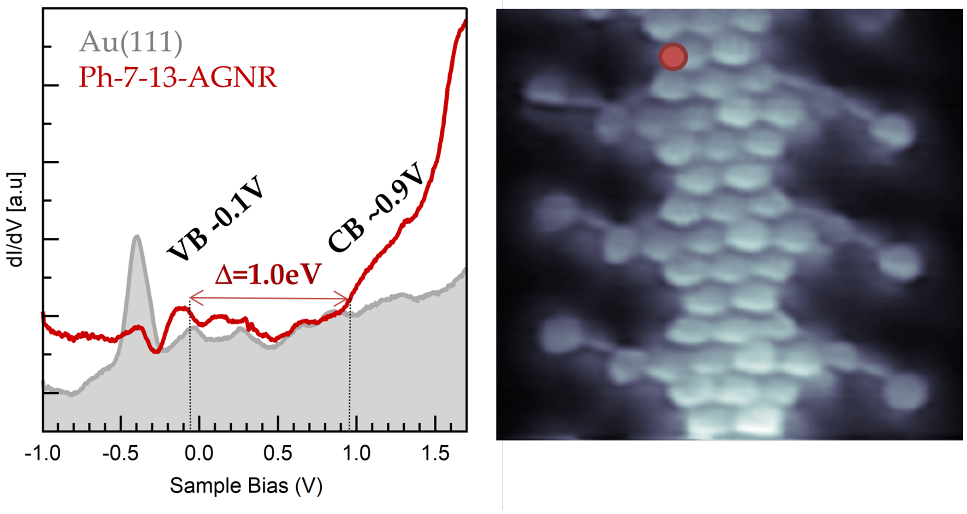


Fig. S7: Electronic properties of the *Ph*-7-13-AGNR. dI/dV spectra acquired at the marked point of the *Ph*-7-13-AGNR displayed in the STM image of Fig. 1c. The onset of the CB and VB bands can be identified in the spectrum (red line). The reference spectra acquired on Au(111) is added in shaded gray. dI/dV spectra was acquired using the lock-in technique, with a modulation voltage of 30 mV and a frequency of 2.5 kHz.

# 4 Surface-induced planarization of the peripheral phenyl ring in isolated ribbons

For the surface supported calculations to study the planarization of the peripheral phenyl ring, we employed a supercell consisting of 4 layers of a Au(111) $8 \times 6$ unit cell with a structure formed by 16 C and 12 H atoms placed on top of one of the Au surfaces. The geometry of the overlayer was extracted from the relaxed gas phase structure of *Ph*-7-13-AGNR and the top most layer of Au and the overlayer were relaxed until the forces were less than 0.04 eV/Å. An energy-cutoff of 300 Ry and a single $k$ point ($\Gamma$) were considered. The results summarized in Fig. S8 show how, in the case of individual ribbons, the interaction with the surface dominates over the steric repulsion with the single adjacent ring, resulting in an

effective planarization of the structure. This is in contrast to that found in the NPG bridges, as reported in Figs. 2c and d, where the surface-induced planarization is counteracted by the steric repulsion from the two adjacent rings.

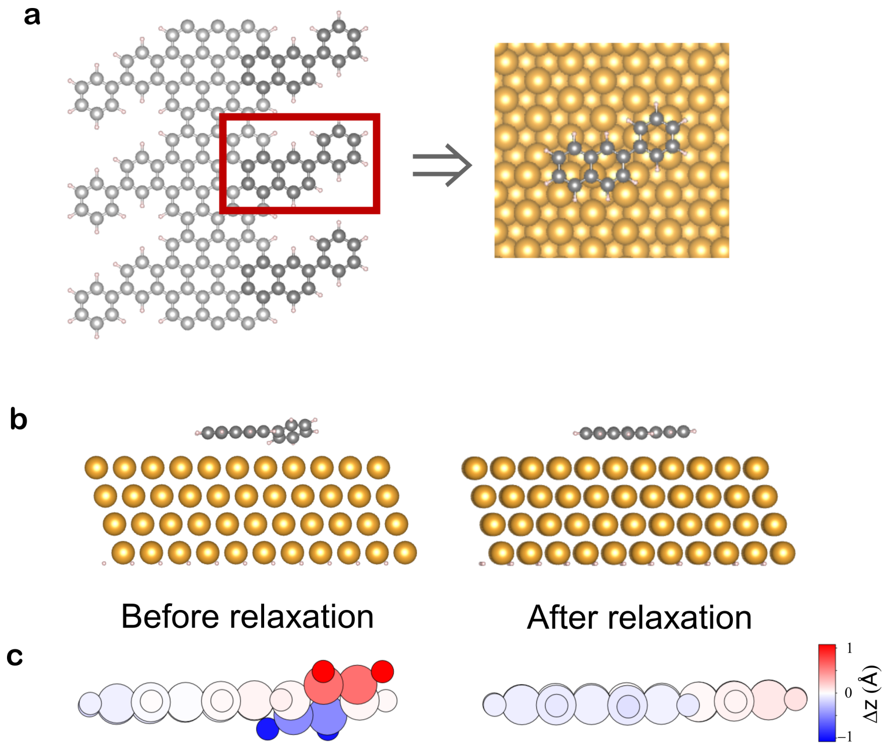


Fig. S8: **a**, Schematics of the atomic structure of *Ph*-7-13-AGNR (left). In order to reduce the computational cost, a representative section, highlighted with a red rectangle, has been used in the relaxation study on the Au(111) surface (right). **b**, Side view of the structure before (left) and after (right) relaxation on the Au(111) surface. The twisted initial conformation (left) corresponds to that of the relaxed free-standing structure. **c**, Same structures as those represented in **b**, with the color code indicating the out-of-plane deviation from the average plane (Δz). The substrate has been removed for clarity.

# References

(1) de Oteyza, D. G.; García-Lekue, A.; Vilas-Varela, M.; Merino-Díez, N.; Carbonell-Sanromà, E.; Corso, M.; Vasseur, G.; Rogero, C.; Guitián, E.; Pascual, J. I.; Ortega, J. E.; Wakayama, Y.; Peña, D. Substrate-Independent Growth of Atomically Precise Chiral Graphene Nanoribbons. *ACS Nano* **2016**, *10*, 9000–9008.

(2) Soler, J. M.; Artacho, E.; Gale, J. D.; García, A.; Junquera, J.; Ordejón, P.; Sánchez-Portal, D. The SIESTA Method for Ab Initio Order-N Materials Simulation. *J. Phys.: Condens. Matter* **2002**, *14*, 2745.

(3) García, A. et al. Siesta: Recent developments and applications. *The Journal of Chemical Physics* **2020**, *152*, 204108.

(4) Troullier, N.; Martins, J. L. Efficient pseudopotentials for plane-wave calculations. *Phys. Rev. B* **1991**, *43*, 1993–2006.

(5) Junquera, J.; Paz, O.; Sánchez-Portal, D.; Artacho, E. Numerical atomic orbitals for linear-scaling calculations. *Phys. Rev. B* **2001**, *64*, 235111.

(6) Perdew, J. P.; Burke, K.; Ernzerhof, M. Generalized Gradient Approximation Made Simple. *Phys. Rev. Lett.* **1996**, *77*, 3865–3868.

(7) Monkhorst, H. J.; Pack, J. D. Special points for Brillouin-zone integrations. *Phys. Rev. B* **1976**, *13*, 5188–5192.

(8) Papior, N.; Lorente, N.; Frederiksen, T.; García, A.; Brandbyge, M. Improvements on non-equilibrium and transport Green function techniques: The next-generation transiesta. *Computer Physics Communications* **2017**, *212*, 8–24.

(9) Papior, N. sisl: v0.12.2. 2021; `https://doi.org/10.5281/zenodo.597181`.

(10) Calogero, G.; Papior, N. R.; Kretz, B.; Garcia-Lekue, A.; Frederiksen, T.; Brandbyge, M. Electron Transport in Nanoporous Graphene: Probing the Talbot Effect. *Nano Letters* **2019**, *19*, 576–581.

(11) Calogero, G.; Alcón, I.; Papior, N.; Jauho, A.-P.; Brandbyge, M. Quantum Interference Engineering of Nanoporous Graphene for Carbon Nanocircuitry. *Journal of the American Chemical Society* **2019**, *141*, 13081–13088.

(12) Moreno, C.; Panighel, M.; Vilas-Varela, M.; Sauthier, G.; Tenorio, M.; Ceballos, G.; Peña, D.; Mugarza, A. Critical Role of Phenyl Substitution and Catalytic Substrate in the Surface-Assisted Polymerization of Dibromobianthracene Derivatives. *Chemistry of Materials* **2018**, *31*, 331–341.
